# Delayed Diagnosis and Multimodality Management of a Primary Myxoid Chondrosarcoma of the Skull Base: A Case Report

**DOI:** 10.7759/cureus.113027

**Published:** 2026-07-20

**Authors:** Mynor Oswaldo Quiroa Rodriguez, Kenny Mardoqueo Rojas Natareno

**Affiliations:** 1 General Surgery, Instituto Guatemalteco de Seguridad Social, Guatemala City, GTM; 2 Neurological Surgery, Instituto Guatemalteco de Seguridad Social, Guatemala City, GTM

**Keywords:** complex surgical resection, myxoid chondrosarcoma, parasellar tumors, sellar tumor, skull base tumors

## Abstract

A myxoid chondrosarcoma involving the skull base is an uncommon malignant tumor that can closely resemble more frequent sellar and parasellar lesions, making diagnosis and treatment challenging. We report the case of a 41-year-old man who presented with progressive headache and visual impairment and was found to have a large skull base mass extending into adjacent intracranial compartments with compression of the optic pathway. Owing to the tumor’s location and extensive neurovascular involvement, maximal safe resection was performed through a transcranial approach. Histopathologic findings favored a diagnosis of a myxoid chondrosarcoma, although immunohistochemical and molecular characterization could not be performed because of resource limitations. Histologic grade, Ki-67 proliferation index, and ancillary pathological studies were not assessable because of limited diagnostic resources. The postoperative course was complicated by transient diabetes insipidus, which responded to medical treatment, and adjuvant radiotherapy was administered for the residual tumor. This case highlights the importance of considering myxoid chondrosarcomas in the differential diagnosis of atypical sellar and parasellar masses and emphasizes the role of individualized surgical planning combined with adjuvant radiotherapy to optimize management when complete resection is not feasible.

## Introduction

Chondrosarcomas are rare, malignant cartilaginous neoplasms of the skull base that make up 6% of all skull base cancers and 0.15% of all intracranial neoplasms. The rare form of chondrosarcomas is the myxoid type, characterized by the presence of a rich chondromyxoid matrix and the occurrence of tumor cells that are similar to the chondrocytes [1,2]. Skull base chondrosarcomas usually arise from the joints (synchondroses) of the spheno-occipital, sphenopetrosal, or petroclival regions. The sellar and parasellar area is a very challenging area of surgery where all the important neurovascular structures such as the optic apparatus, cavernous sinus, internal carotid artery, and hypothalamic/pituitary axis are located [3,4]. In the event of a lesion located in the centre of the sellar region, these conventional sellar approaches (transsphenoidal, sublabial, or endoscopic endonasal) tend to be the best options. However, transcranial techniques may be necessary for sufficient decompression in tumors that have suprasellar, frontotemporal, and/or intraventricular extension [5,6]. A case with a stellar primary myxoid chondrosarcoma with significant extracapsular disease that involved the optic nerves, progressed to profound visual impairment, became obstructive hydrocephalic due to involvement, and had a residual disease after maximal safe resection is presented. The case highlights the decision-making process of neurosurgery for tumors that do not lend themselves to the usual sellar approach and shows the difficulty in diagnosing unusual cartilaginous tumors of the skull base that mimic more common tumors.

## Case presentation

Patient information and history

A 41-year-old single man from Guatemala City developed a two-year history of progressive headache and subsequently decreased vision in his right eye, which ultimately progressed to the left eye. He had a past medical history of hypothyroidism for which he took levothyroxine (100 mcg half tablet every 48 hours) for two years from the time of diagnosis. He stated that he had no history of previous surgery, allergies, familial history of any significance, drinking alcohol, smoking tobacco, or using drugs.

Initial presentation

The patient was referred for hospital admission on Day 1 due to a sellar-region mass and progressive visual symptoms. The primary admission history documented an approximately two-year history of headache, followed by loss of vision in the right eye with later progression to involve the left eye. A preoperative consultation noted difficulty focusing with the right eye. Working differential diagnoses included optic chiasm lesion, compressive lesion, neoplastic lesion, pituitary adenoma, meningioma, macroadenoma, and atypical glioma. Clinical impression documented a tumor in the sellar region, loss of vision in the right eye, and hypothyroidism. Formal visual acuity was not measured in the available clinical record. The clinical record from the patient's oncohaematologic evaluation confirmed that he was haemostatically and circulatorially stable (BP = 122/74 mmHg, HR = 83 bpm, RR = 14 b/m, SpO₂ = 96%). He demonstrated evidence of being awake, moving around, and oriented to time/place/person. His pupils were equal in size and responsive to light. He had esotropic eyes. Examination of his motor system indicated there were no limitations or abnormalities detected regarding the movement of the upper/lower limbs bilaterally. No documentation existed for any specific focal neurological deficit. His abdomen was soft and exhibited no evidence of abdominal wall pain or other signs of peritonitis.

Preoperative diagnostic workup

Visual field assessment was performed at the initial presentation. The 60-degree campimetry demonstrated bilateral abnormalities. The right eye showed fixation loss 0/11, false positives 0%, diffuse absolute scotomas dispersed throughout the visual field, and visual field constriction. The left eye demonstrated fixation loss 0/18, false positives 2%, dispersed relative and absolute scotomas, and visual field constriction. The examination was concluded as abnormal in both eyes, objectively confirming bilateral visual pathway compromise.

Initial preoperative MRI

**Figure 1:**
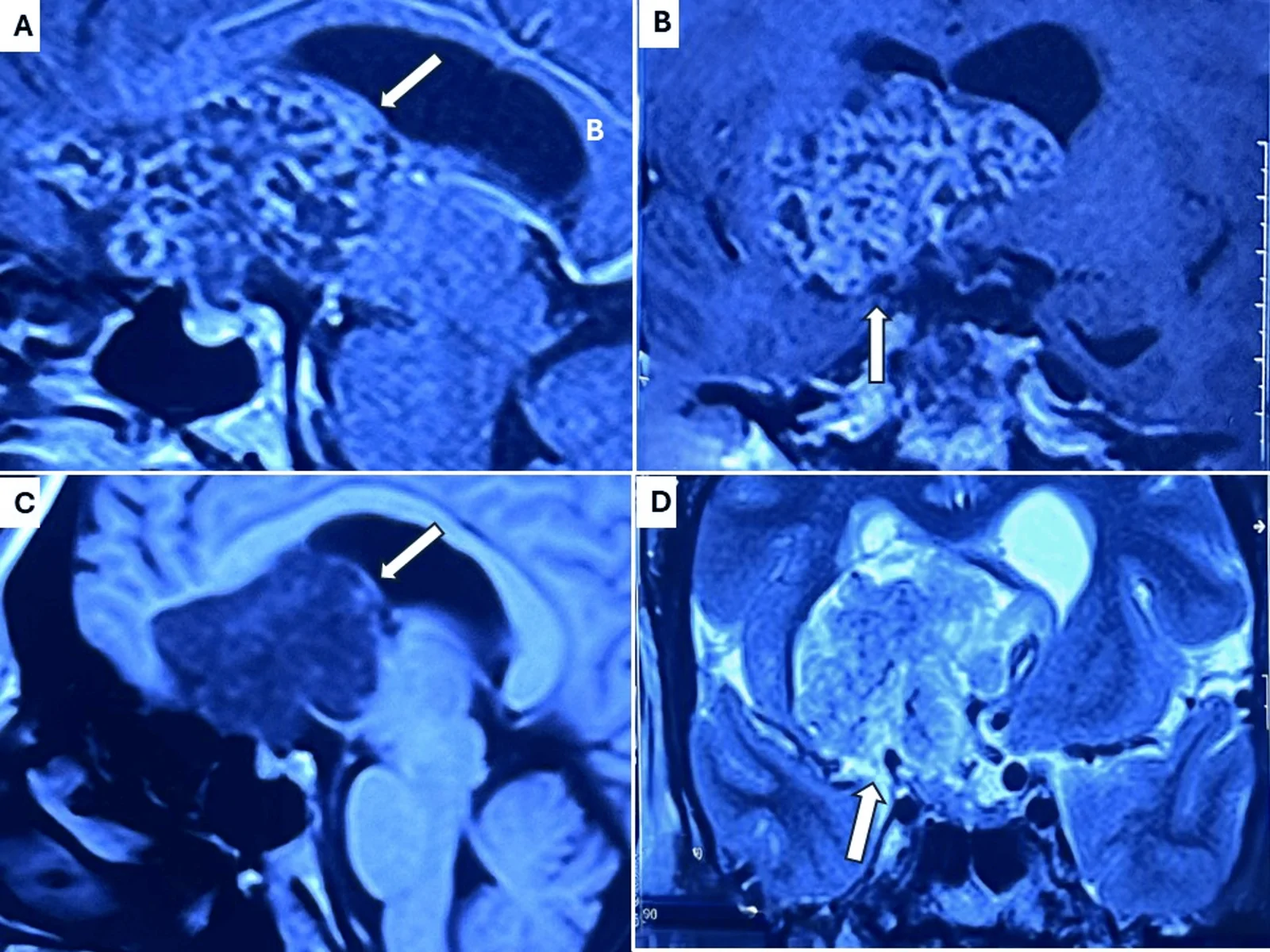
Preoperative magnetic resonance imaging demonstrating a large parasellar/suprasellar mass with superior extension and mass effect on adjacent structures (A) Sagittal preoperative MRI showing the lobulated lesion extending superiorly from the sellar/parasellar region, with associated compression and displacement of the ventricular system (arrow). (B) Coronal preoperative MRI demonstrating the superior parasellar/suprasellar extent of the mass and its inferior attachment/continuity toward the sellar region (arrow). (C) Additional sagittal preoperative MRI view highlighting the superior tumor margin and marked cephalic growth with distortion of adjacent intracranial anatomy (arrow). (D) Coronal preoperative MRI showing the lesion occupying the parasellar/suprasellar compartment, with an inferior relationship to the sellar region (arrow) and an associated mass effect on the hypothalamic-chiasmatic region and ventricular system.

Sagittal and coronal magnetic resonance imaging (MRI) demonstrated a large parasellar mass. The large mass, measuring about 65 x 60 mm, was found on sagittal and coronal MRI superior to and to the right of the sella turcica. The lesion was hypointense on T1-weighted and hyperintense on T2-weighted MR images and showed central and peripheral areas of necrosis and heterogeneous central and peripheral enhancement on contrast enhancement studies. It had cephalic and rostral extensions and thus compression of the hypothalamic region, right optic chiasm, third ventricle, and lateral ventricles. The pituitary was small, but the C3-4 sections of the internal carotids, clinoid, and pterygoid processes were all normal, as was the sphenoid sinus. No sign of an intralesional hemorrhage was found. Finally, the imaging results were compatible with a large parasellar mass, resulting in significant compression of the optic chiasm (Figure 1).

Repeat preoperative MRI

**Figure 2:**
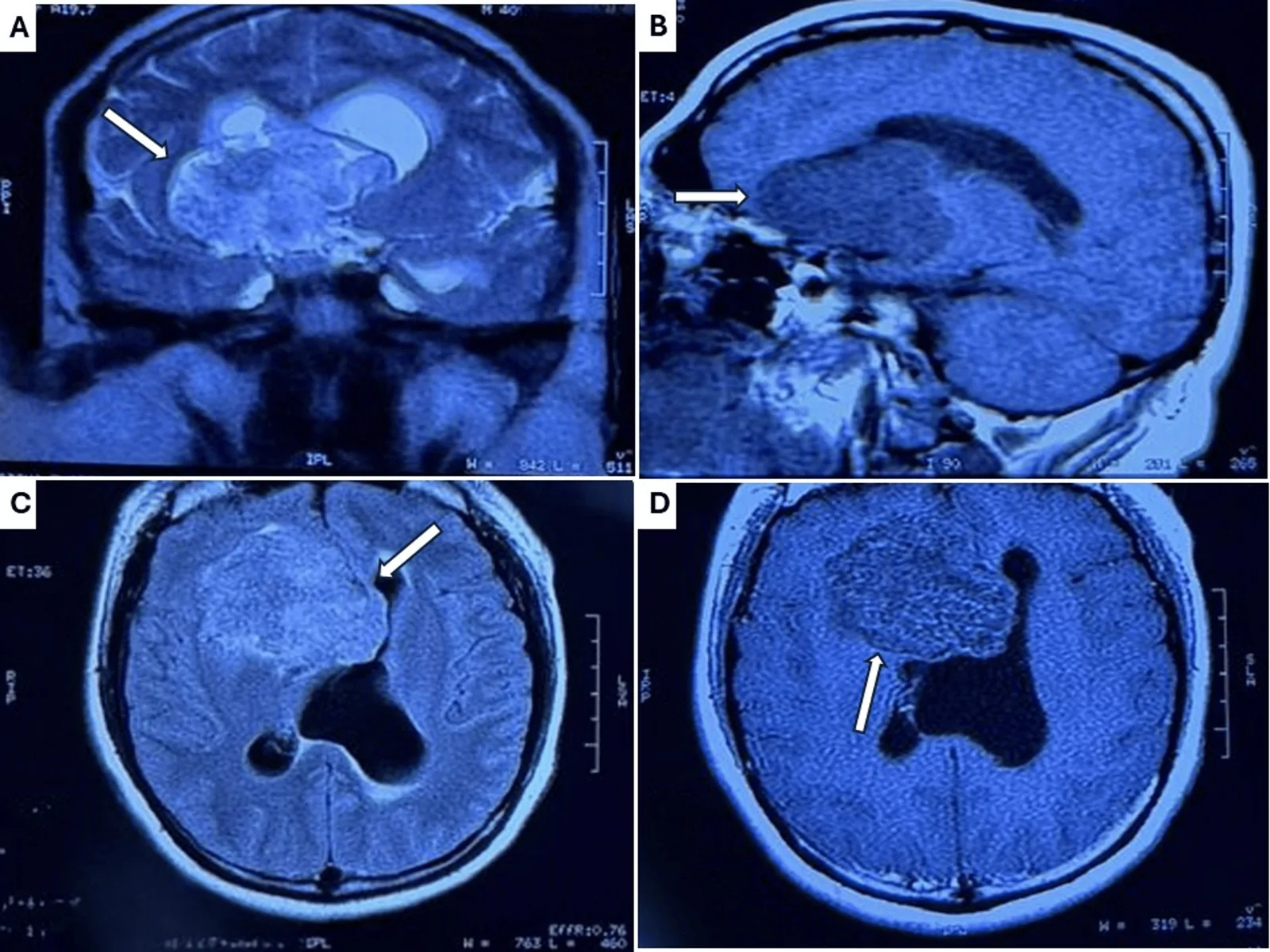
Preoperative MRI demonstrating a large heterogeneous mass centered near the interhemispheric fissure with a marked mass effect and obstructive hydrocephalus (A) Coronal T2-weighted image of the large multinodular lesion, with a heterogeneous internal signal, adjacent to the interhemispheric fissure and a compression of the anterior ventricular system and associated hydrocephalus (arrow), mainly involving the left lateral ventricle. (B) Sagittal T1-weighted image demonstrating the lesion projecting anteriorly toward the genu of the corpus callosum and inferiorly toward the skull base/sellar region (arrow). (C) Axial T1-weighted image showing a right-predominant mass effect with obliteration of the frontal horns and displacement/compression of the ventricular system, as well as associated bilateral cerebral edema (arrow). (D) Axial T1-weighted image demonstrating the superior aspect of the mass and its close relationship to the foraminal region of Monro, contributing to obstructive hydrocephalus (arrow).

Coronal, sagittal, and axial MRI demonstrated a marked mass effect and obstructive hydrocephalus before surgery. MRI demonstrated a large heterogeneous multinodular mass adjacent to the interhemispheric fissure, projecting toward the genu of the corpus callosum and extending toward the right optic foramen. The size of the lesion was estimated as about 58 mm × 58 mm but later as 62 mm × 51 mm. It was demonstrated to have patches of both hypo- and hyperintense signals on T2-weighted images, a hypointense signal on sagittal T1-weighted images, and heterogeneous enhancement, with a hyperintense peripheral rim around a partially necrotic core area. Considerable mass compression of the anterior ventricular system caused obliteration of the frontal horns and obliteration of the right lateral ventricle, along with obstruction of the foramina of Monro, and became associated with obstructive hydrocephalus, mainly in the left lateral ventricle. There was also diffuse bilateral cerebral edema. The possible differential diagnoses in the radiologic appearance were glioblastoma and atypical meningioma. Other incidental findings were hypertrophy of the nasal turbinates with partial airway obstruction, a left maxillary mucous retention cyst, and a right maxillary mucocele, with no evidence of secondary right proptosis. The imaging findings were felt to be nondiagnostic, and histopathologic correlation was suggested for final diagnosis (Figure 2).

Overall, the lesion was described as a large heterogeneous mass measuring approximately 58 × 58 mm and 62 × 51 mm in different imaging planes and phases, with a partially necrotic center, heterogeneous signal characteristics, dispersed contrast enhancement, and projection toward the right optic foramen. The imaging findings presented across all figures correspond to the same lesion. Before surgery, the radiologic differential diagnosis was glioblastoma versus atypical meningioma; however, definitive histopathological examination of the resected specimen subsequently confirmed the diagnosis of conventional chondrosarcoma.

Histopathological findings

**Figure 3:**
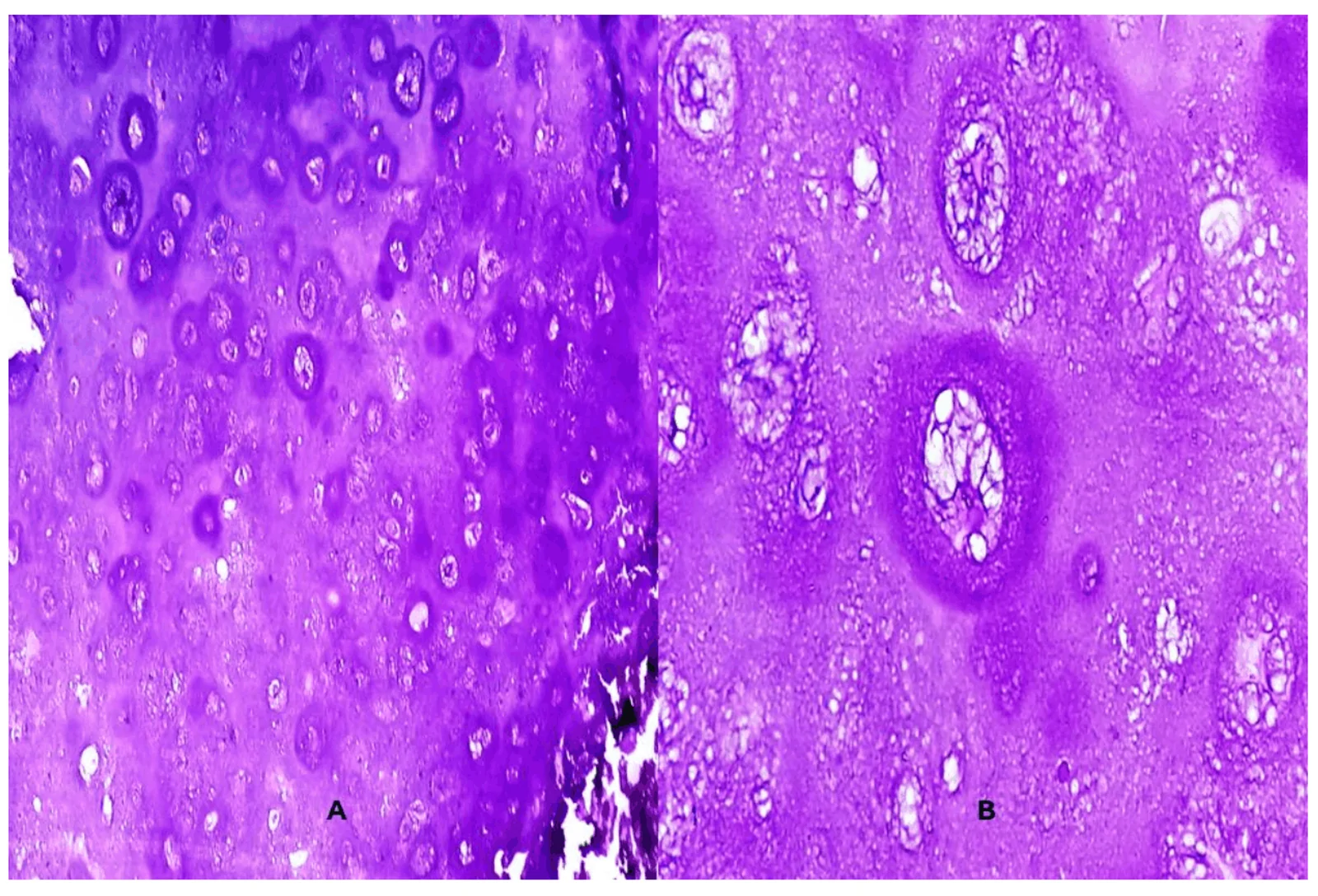
Histopathological findings of the myxoid chondrosarcoma (A) Low-power view showing multiple irregular fragments of chondroid tissue embedded in abundant basophilic chondromyxoid matrix. (B) Higher-magnification view demonstrating rounded lacunar spaces with chondrocyte-like tumor cells and focal myxoid/vacuolated areas. These findings are consistent with the pathological diagnosis of a myxoid chondrosarcoma in the resected right sellar/frontal tumor specimen.

The anatomical pathology report documented one specimen weighing 49 grams with aggregate dimensions of 9 × 8.2 × 2 cm. Gross examination revealed multiple gray tissue fragments with violaceous and white areas; firm, chondroid irregular tissue; and additional hard fragments up to 0.4 × 0.3 × 0.2 cm. Six cassettes were submitted (Figure 3).

Final diagnosis: right sellar and frontal tumor, resection-myxoid chondrosarcoma

The available pathology documentation was limited to the gross specimen description and the final histopathologic diagnosis. Histologic grading, immunohistochemistry, molecular profiling, and margin assessment were not available because of limited local diagnostic resources. Consequently, the diagnosis was based on routine histopathologic evaluation alone, and ancillary studies that could further support the diagnosis and exclude histologic mimics were not performed. Routine preoperative infectious disease screening, including HIV and hepatitis B/C serology, was performed according to institutional protocol. The results did not influence the diagnosis or management of the intracranial lesion but are reported for completeness (Table 1).

**Table 1:** Preoperative laboratory studies BUN: Blood urea nitrogen; TSH: thyroid-stimulating hormone; INR: international normalized ratio; aPTT: activated partial thromboplastin time; LH: luteinizing hormone; FSH: follicle-stimulating hormone; ACTH: adrenocorticotropic hormone

Parameter	Patient's value	Reference range
Coagulation profile		
PT	12.5 sec	11–14 sec
INR	1.10	0.8–1.2
aPTT	33.5 sec	25–35 sec
Thrombin time	13.3 sec	14–21 sec
Complete blood count		
WBC	6.08 × 10³/µL	4.0–10.0 × 10³/µL
Hemoglobin	15.7 g/dL	13.5–17.5 g/dL
Hematocrit	49.0%	41–53%
Platelets	276 × 10³/µL	150–400 × 10³/µL
Serum chemistry		
Creatinine	0.85 mg/dL	0.7–1.3 mg/dL
BUN	20.0 mg/dL	7–20 mg/dL
Sodium	141 mmol/L	135–145 mmol/L
Potassium	3.8 mmol/L	3.5–5.0 mmol/L
Calcium	9.8 mg/dL	8.5–10.5 mg/dL
Magnesium	2.37 mg/dL	1.7–2.4 mg/dL
Phosphorus	3.5 mg/dL	2.5–4.5 mg/dL
Glucose	79 mg/dL	70–99 mg/dL (fasting)
Endocrine profile		
Prolactin	12.3 ng/mL	4.0–15.2 ng/mL
Cortisol (before 10:00 h)	254 µg/dL†	5–25 µg/dL (morning)
Testosterone	1.46 ng/mL	2.8–8.0 ng/mL (280–800 ng/dL)
TSH	3.01 µIU/mL	0.4–4.5 µIU/mL
Total T3	0.97 ng/mL	0.8–2.0 ng/mL
Total T4	10.37 µg/dL	4.5–12.5 µg/dL
Free T4	1.02 ng/dL	0.8–1.8 ng/dL
Growth hormone	0.0467 ng/mL	<5.0 ng/mL
LH	4.29 mIU/mL	1.7–8.6 mIU/mL
FSH	7.45 IU/L	1.5–12.4 IU/L
ACTH	Not available	7.2–63.3 pg/mL (morning)
Infectious serology	Result	
HIV	Nonreactive	—
HBsAg	Nonreactive	—
Anti-HBs	Positive (46.3 IU/L)	—
Anti-HBc total	Positive (2.76 index)	—
Anti-HBc IgM	Nonreactive	—
Anti-HBe	Positive (1.59)	—
HBeAg	Nonreactive	—
Anti-HCV	Nonreactive	—

Preoperative neurosurgical evaluation

A preoperative neurosurgical consultation described the lesion as extending toward the third ventricular recess with right frontal and temporal involvement. Critically, the consultation documented that the sella turcica anatomy was not favorable for a conventional sellar approach, leading to consideration of a transcranial approach. This represented a defining neurosurgical element of the case, as tumor anatomy and extension supported a cranial route rather than a simple sellar approach. The patient was considered a surgical candidate and referred for endocrine preoperative assessment.

Neurosurgical intervention

The patient underwent a right frontal craniotomy for the removal of a sellar-region tumor utilizing selective hemostasis and a Jackson Pratt drain. This was done under general anesthesia. After the patient was anesthetized, he was placed into position for a right frontal transcranial approach. The area around the surgical site was prepared and draped with standard sterile technique. A right hemicoronal incision was made to allow good anterior cranial exposure. This incision went through the skin, subcutaneous fat, aponeurosis, and finally the muscles. During this time, adequate hemostasis of the scalp and muscle layers was obtained. Once adequate exposure of the right frontal bone had been obtained, a right frontal craniotomy was performed using a Midas Rex trephine. By removing the bone flap, access to the intracranial compartment was obtained along with access to a surgical corridor to the sellar/suprasellar region. Upon completion of the craniotomy and exposure, it became apparent that there was a solid, well-vascularized tumor located in the sellar/suprasellar region extending greater than one-half from the midline on each side. The tumor measured about 5 cm (two inches) at its greatest diameter. Due to these findings, the surgeon attempted to remove the tumor as much as possible while obtaining safe hemostasis. To achieve bone hemostasis, bone wax was used. Then surgery was applied to the wound bed. Finally, after irrigation with saline solution, the bone flap was returned to its original location and fastened to the surrounding bone with cranial fixation devices. Closure of the soft tissues was accomplished with a Vicryl 2-0 suture. A Jackson-Pratt drain was placed and anchored to the soft tissue with a silk 2-0 suture. Staples were utilized for closing the skin. There were no complications noted during the case.

Surgical complexity and rationale for residual disease

**Figure 4:**
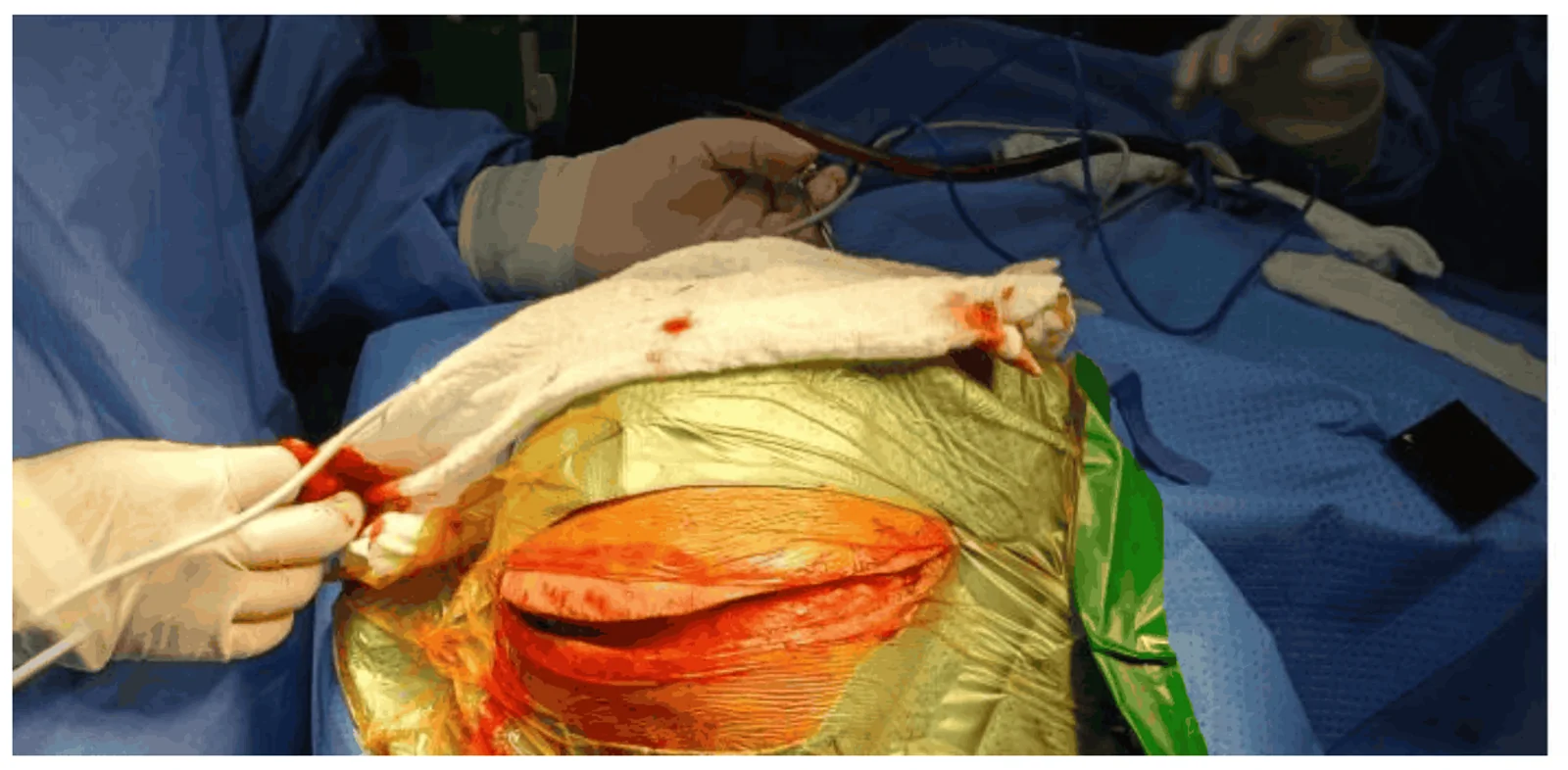
Intraoperative exposure through right hemicoronal incision for transcranial approach to a sellar/parasellar mass Photograph obtained during surgery showing the right hemicoronal incision and surgical exposure used to access the lesion through a transcranial approach.

**Figure 5:**
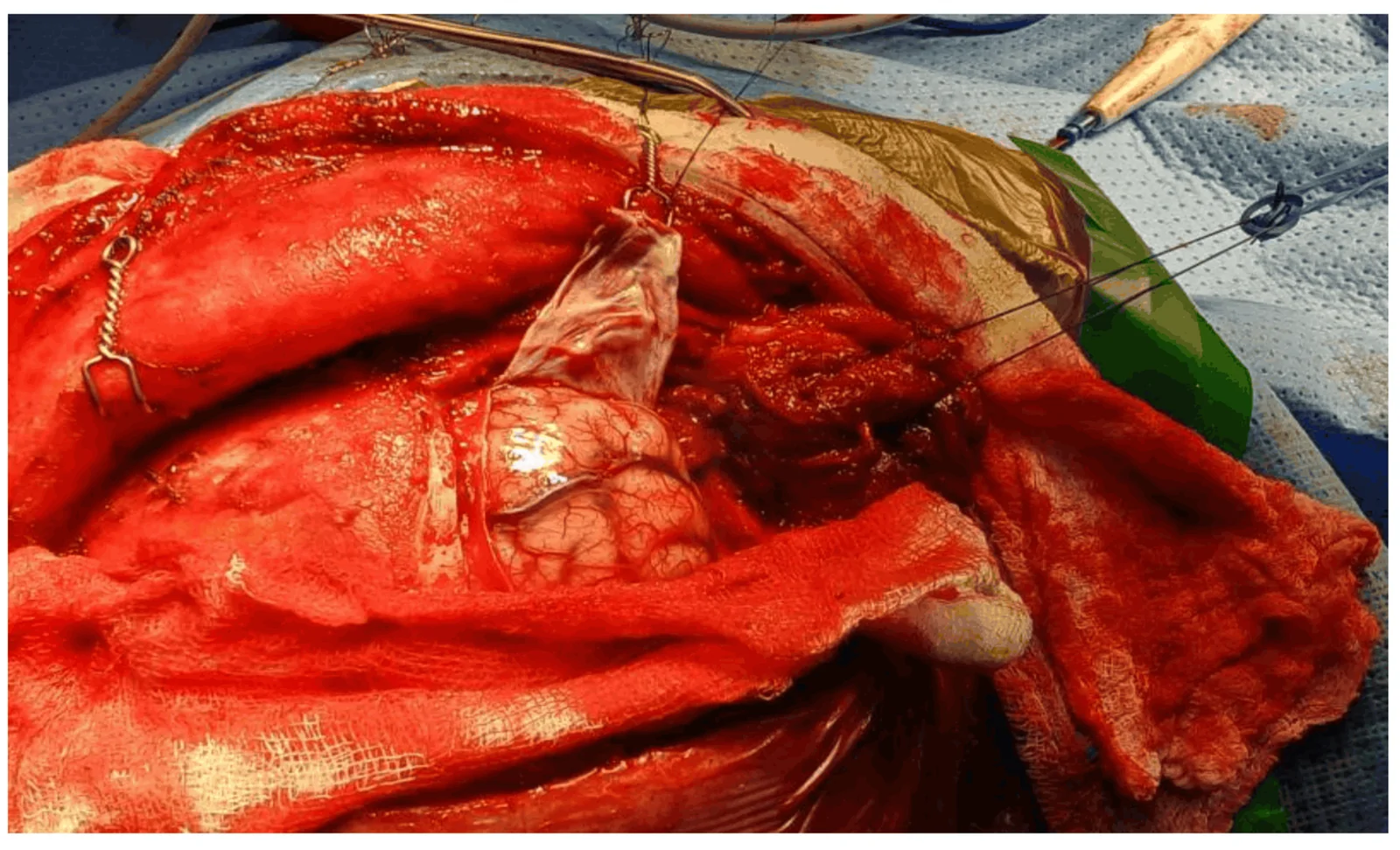
Intraoperative view of tumor exposure during right frontal craniotomy Photograph obtained during surgery showing intracranial exposure and direct visualization of the lesion during transcranial resection.

**Figure 6:**
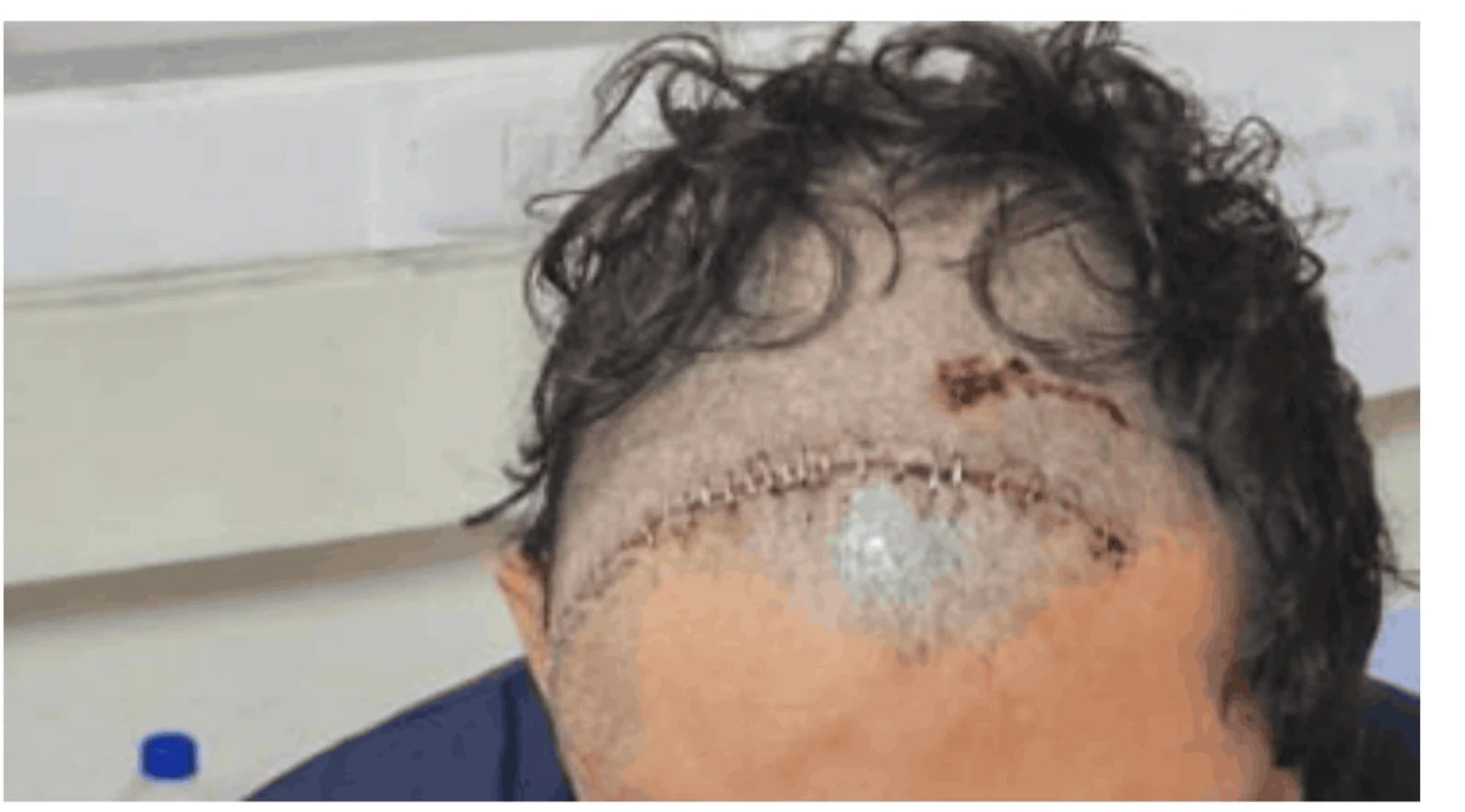
Postoperative wound appearance following the right hemicoronal craniotomy approach Photograph demonstrating the postoperative scalp incision closed with staples after right frontal transcranial surgery for lesion resection.

Intraoperatively, the lesion was described as a solid frontotemporoparietal endocranial tumor with a cartilaginous appearance and vascularity. These tumor-vascular relationships were complex; the carotid artery was surrounded at segment 6, the pericallosal arteries were involved, and there was displacement but not deformity of the ophthalmic artery. An association with the neighboring brain parenchyma made the mobilization of the tumor difficult. Controlled internal debulking of this solid cartilaginous tumor was restricted by the vascularity of this growth and by the lack of an ultrasonic aspirator during surgery. To this end, resection was done following the maximally safe resection principles with a focus on decompression and the reduction of tumor burden without any vascular or neural injury. This anatomical and technical context should be taken into account when considering the residual postoperative disease (Figures 4-6).

Immediate postoperative course

At six hours after surgery, the patient had an increased urine output (4 mL/kg/hour), decreased urine specific gravity (1.003), and serum sodium levels greater than 135 mEq/L, indicating central diabetes insipidus, for which intravenous desmopressin was administered. Monitoring was followed thereafter, and the urine output was at less than 2 mL/kg/hour and, subsequently, 0.60 mL/kg/hour with regular doses of desmopressin. The hospitalization records show that desmopressin was discontinued after the patient had the first 48 hours with no need for it and was able to get his urine output back to normal before being discharged. InScribe sent a case to the internal medicine team to be followed up on.

There were no signs of oozing blood; the patient cited being in “good health” and not being on a vasopressor, being afebrile and neurologically intact, and tolerating oral feeding. Post-surgical antibiotic coverage was given as ceftriaxone, 2 g, via intravenous drip once, and vancomycin as 500 mg per dose (at 72 kg weight, 15 mg per kg per dose) for seven days. The Karnofsky Performance Status (KPS): the 48-hour KPS score was 60, and the fifth postoperative day score was 90.

Early postoperative MRI (three months after surgery)

**Figure 7:**
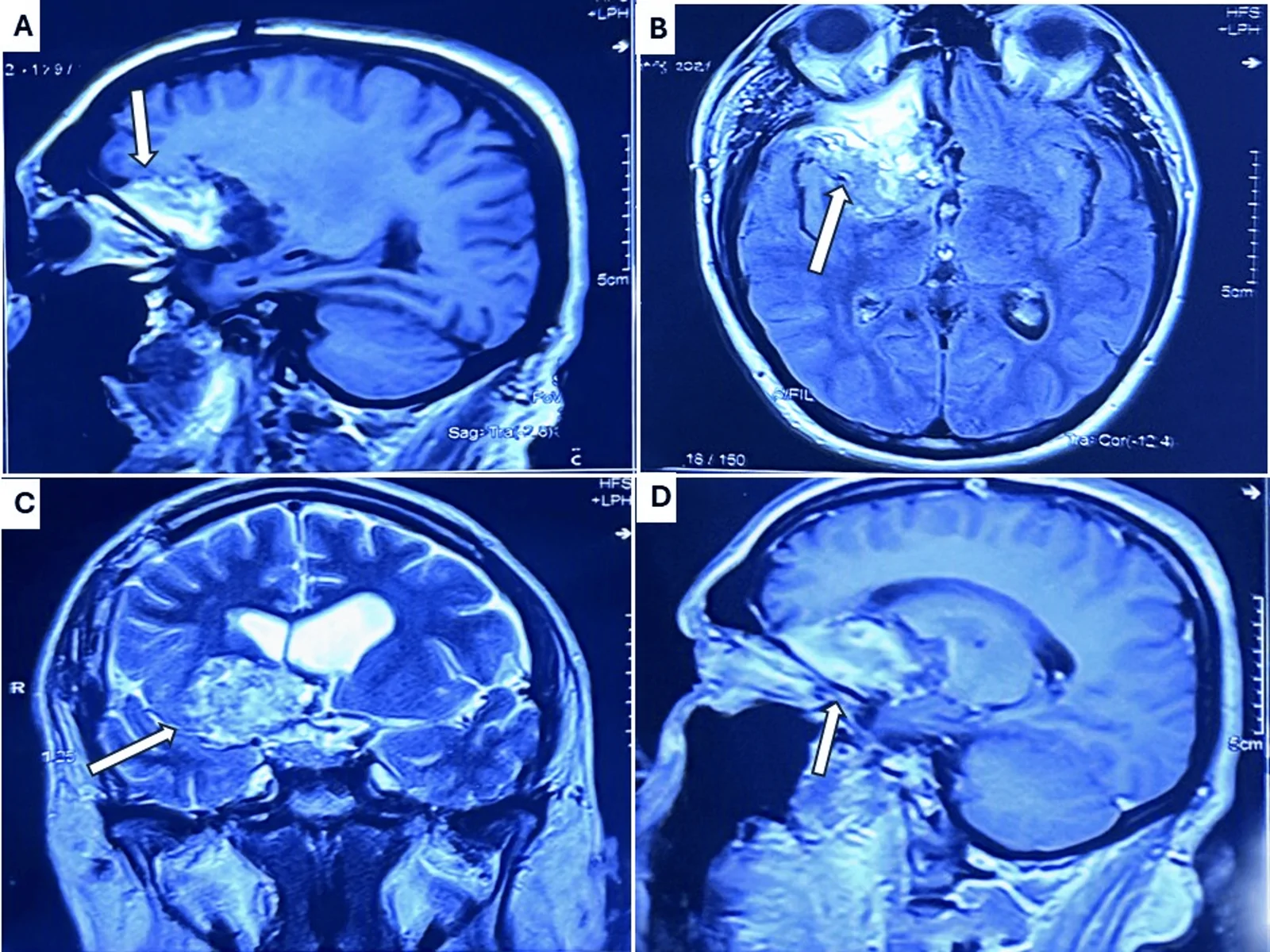
Postoperative magnetic resonance imaging demonstrating a persistent right temporal lesion radiologically interpreted as probable residual disease. (A) Sagittal T1-weighted image showing persistent abnormal tissue in the postoperative right anterior skull-base/temporal region (arrow). A residual right temporal lesion with a local mass effect is seen in (B), an axial T1-weighted image. (C) Coronal T2-weighted: The lesion appears to be heterogeneous and is about 45 x 40 mm with edema around the lesion (arrow). (D) Sagittal contrast-enhanced T1 magnetic resonance imaging showing enhancing postoperative residual tissue through the operative area (arrow).

Sagittal, axial, and coronal MRI demonstrated residual disease measuring approximately 45 × 40 mm. MRI revealed a hyperintense lesion in the right temporal lobe on FLAIR and T2-weighted sequences, which was approximately 45×40 mm in size. There was surrounding edema and a halo, which was non-enhancing, indicating a possible previous hemorrhage. The lesion post-contrast administration was believed to be residual tissue of the previously identified primary lesion. It has had a mass effect due to extrinsic compression of the right middle cerebral artery, the frontal horn of the right lateral ventricle, the genu of the corpus callosum, and the optic nerve of the same side. The degree of dilatation of the right lateral ventricle was also noticed. The remainder of the midbrain, pons, basal ganglia, cerebral hemispheres, pituitary gland, optic chiasm, tuber cinereum, fourth ventricle, cisterna magna, and the 7th and 8th nerve complexes were otherwise normal. Overall, the imaging findings were consistent with a residual right temporal lesion complicated by ongoing mass effect and/or postoperative changes, with additional incidental findings of bilateral turbinate hypertrophy, nasal septal deviation to the left, and a left maxillary retention cyst measuring 20 × 20 mm (Figure 7).

The latter study revealed disturbance of the adjacent ventricular structures characterized by a non-enhancing halo suggestive of old hemorrhagic change with a persistent mass effect.

Adjuvant radiotherapy

Surgery and postoperative imaging results were consistent with residual disease, and the patient underwent adjuvant radiotherapy. Treatment details: a linear accelerator was used with an energy of 6 MV of photons, 23 sessions of 2 Gy fractionated treatment, and the conformal fields technique for the tumor bed. Treatment positioning was done in the supine position with the aid of a custom-made thermoplastic immobilization mask; with the aid of CT-based simulation, onboard CBCT verification was carried out. No adverse events were reported on the available radiotherapy report, and tolerance was acceptable.

Late follow-up MRI (eight months after surgery)

**Figure 8:**
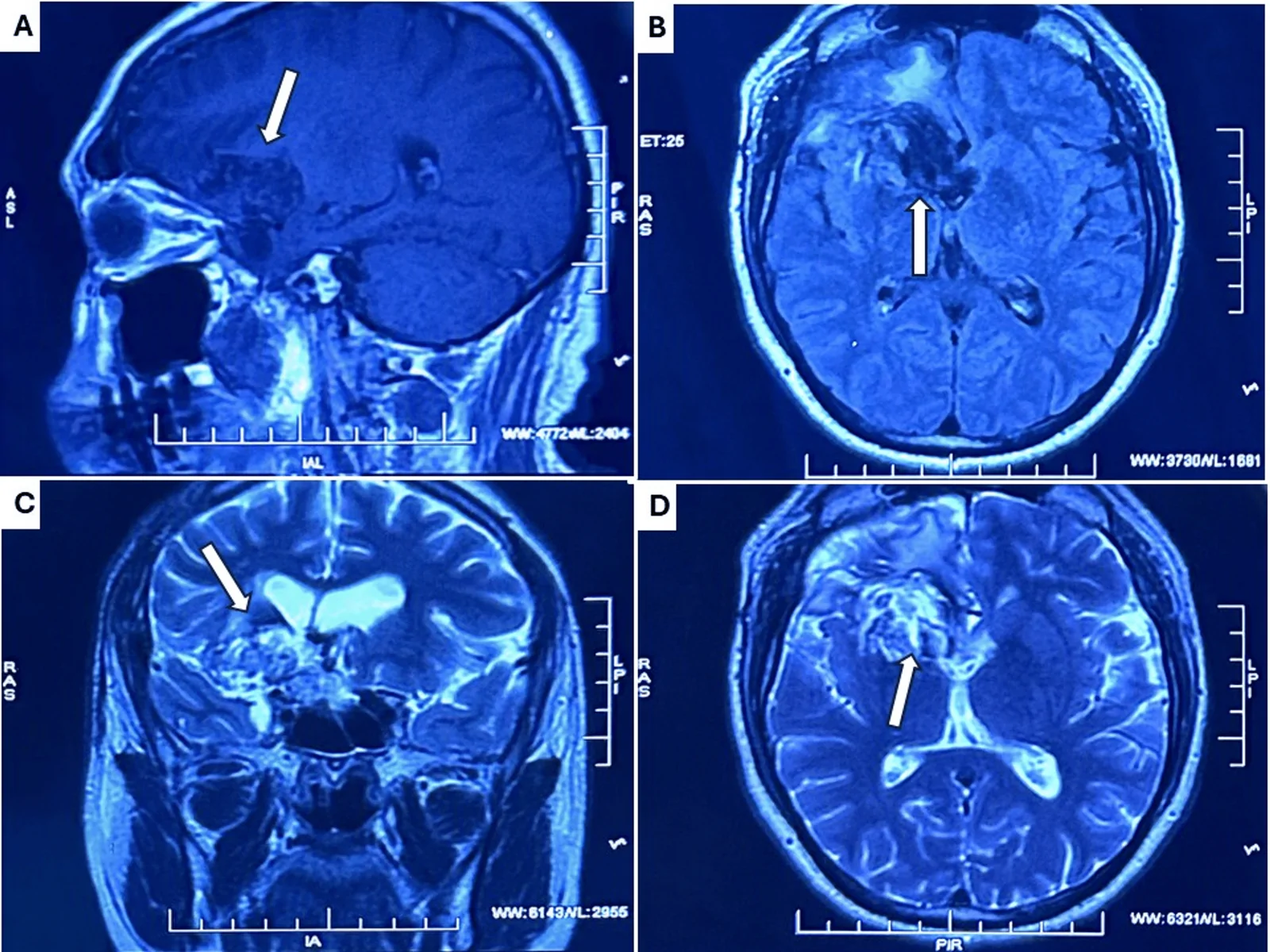
Postoperative right frontal-temporal gliotic/encephalomalacic change and a small residual lesion anterior and to the right of the sella turcica identified on MRI follow-up imaging (A)Sagittal T1-weighted image showing abnormal nodular tissue in the anterior parasellar area which persisted (arrow). (B) Pre-operative T1-weighted image of the same patient after surgery in which there is encephalomalacic-gliotic change in the right frontal/temporal lobe with residual tissue in the parasellar/interhemispheric region (arrow). Coronal T2-weighted image (C) demonstrates a round residual lesion (arrow) next to the dilated ventricular system. The residual lesion with surrounding postoperative parenchymal change is well seen in this axial T2-weighted image (D).

Sagittal, axial, and coronal views showing reduced residual disease (approx. 10x10 mm). There was postoperative gliosis/encephalomalacia, and an enhancing lesion of about 10 × 10 mm in the region of the sella was described as once again being probable residual lesions. The remaining enhancing abnormality was decreased in size compared to the postoperative MRI obtained eight months after surgery. The available imaging did not show a progression, with no documentation of the disease. The MRI revealed macrocystic and microcystic gliosis and encephalomalacia in the right frontal and temporal lobes. The enlarged right lateral ventricle had been identified; there was no evidence of any abnormality in the uninvolved left lateral ventricle. Post-contrast, a rounded, enhancing lesion was seen anterior and right of the sella turcica, which is consistent with the known primary tumor but has now disappeared. Enhancement of the meninges in the anterior interhemispheric sulcus and in the right frontal and parietal areas was noted. The remainder of the CNS (midbrain, pons, corpus callosum, basal ganglia, cerebral peduncles, pituitary gland, optic chiasm, tuber cinereum, fourth ventricle, and cisterna magna) was otherwise unremarkable, as were the paranasal sinuses and globes. Other incidental findings reported were bilateral turbinate hypertrophy, leftward nasal septal deviation, and a left maxillary retention cyst; the latter was 22 × 10 mm in size. Overall, the imaging findings were most consistent with a residual lesion from the previously diagnosed primary tumor, accompanied by postoperative gliotic and encephalomalacic changes. Radiologically, this was thought to be probably residual disease, which was an approximately 10x10 mm lesion (Figure 8).

Follow-up and outcomes

On postoperative day 1, the patient was reviewed by the neurosurgery and oncology teams. Histopathological examination confirmed the myxoid chondrosarcoma, with associated hypothyroidism. Outpatient oncology follow-up, postoperative brain MRI, and further specialist consultations were recommended.

On physical examination, the patient had a blood pressure of 122/74 mmHg, heart rate of 83 beats/min, respiratory rate of 14 breaths/min, and oxygen saturation of 96% on room air. Postoperatively, the patient remained alert and active, with isochoric and reactive pupils, esotropia, a soft and non-tender abdomen, symmetrical movement of all extremities, intact neurological function, orientation to time, place, and person, and no focal neurological deficits. Transient diabetes insipidus developed during the postoperative period and was managed conservatively, with subsequent resolution. Perioperative antibiotics were administered according to the institutional protocol. Details regarding corticosteroid therapy, deep vein thrombosis prophylaxis, intracranial pressure management, anticonvulsant use, structured rehabilitation, and long-term endocrine follow-up were not available in the medical records and therefore could not be included.

Approximately 11 months after surgery, during an oncology follow-up visit, the patient was noted to have residual sellar and right temporal myxoid chondrosarcoma after completing 23 fractions of adjuvant radiotherapy. Although follow-up had been inconsistent, a surveillance brain MRI was planned. Physical examination revealed a blood pressure of 124/89 mmHg, heart rate of 95 beats/min, respiratory rate of 18 breaths/min, oxygen saturation of 95%, body temperature of 36.6°C, weight of 97.5 kg, and height of 165 cm. On the latest follow-up, during a video consultation, the patient reported significant clinical improvement, with complete resolution of headaches and satisfactory wound healing. Visual disturbances persisted but were less severe than at presentation. Although serial brain MRI examinations had been recommended, the patient had not undergone follow-up imaging because of work-related commitments.

At the latest assessment, the patient was alive with persistent residual disease and was not receiving regular specialist follow-up. Available postoperative imaging demonstrated a residual tumor without evidence of documented clinical progression, and the patient reported stable neurological symptoms. The recurrence-free interval was not applicable, as residual disease was present after surgery and there was no documented period of complete disease remission.

## Discussion

The differential diagnosis included chordoma, chondroma, conventional chondrosarcoma, meningioma, solitary fibrous tumor/hemangiopericytoma, esthesioneuroblastoma, and other skull-base sarcomas. Radiologically, the lesion was initially considered most consistent with glioblastoma or atypical meningioma because of its heterogeneous enhancement and aggressive appearance. Histopathologic findings favored a myxoid chondrosarcoma based on the abundant myxoid matrix and cartilaginous differentiation. However, immunohistochemical and molecular studies, which would aid in excluding histologic mimics such as chordoma, chondroid meningioma, and other myxoid neoplasms, were not available because of resource limitations. Therefore, the diagnosis was based on the integration of clinical, radiologic, intraoperative, and routine histopathologic findings.

The case highlights the clinical, radiological, and surgical difficulties associated with a primary myxoid chondrosarcoma of the skull base. The initial presentation with a two-year history of headache and progressive visual loss is typical of sellar/parasellar mass lesions. However, the extensive suprasellar and frontotemporoparietal extension with obstructive hydrocephalus is less common for typical sellar tumors and should raise suspicion for a more aggressive histology. The radiologic differential diagnosis included macroadenoma, meningioma, and glioblastoma. The T2 hyperintensity and necrotic areas are consistent with the myxoid matrix. But there were no specific imaging biomarkers for diagnosis, and this highlighted the need for histopathological confirmation.

Imaging evidence and histopathological confirmation remain key elements in the diagnosis of a primary intracranial myxoid chondrosarcoma (PICMC). Zhu et al. documented the extreme rarity of extraskeletal myxoid chondrosarcomas originating in the central nervous system; they document a cavernous sinus case in their study and conclude that this type of sarcoma should be included in the differential diagnoses for all skull base and parasellar lesions [7]. Qin et al. showed that a PICMC can occur in the posterior fossa, including the cerebellum, and will typically cause sudden deterioration in neurological status due to mass effect, cerebral oedema, or hemorrhage [8]. Earlier, Im et al. demonstrated a primary intraparenchymal myxoid chondrosarcoma that was detached from both the calvaria and meninges, thus extending the range of possible locations of this tumor beyond the common sites at the base of the skull and dura mater. Collectively, it appears that anatomical location does not determine the site of origin and therefore should only be designated as "intracranially" if an extracranial soft tissue or bony lesion cannot be excluded [7-9].

Histopathologic evaluation remains the cornerstone of diagnosis, as imaging findings are suggestive but not definitive. Previous reports have described myxoid chondrosarcomas as comprising well-differentiated round, epithelioid, or spindle-shaped cells arranged in cords and strands within a richly cellular myxoid or myxochondroid matrix, although the degree of vascularization may vary [7-9]. Ancillary immunohistochemical studies are recommended to support the diagnosis, typically demonstrating positivity for S-100 protein and/or vimentin while excluding epithelial, glial, and meningeal differentiation through negative staining for cytokeratin, GFAP, and EMA [7-9]. This immunophenotypic profile is particularly valuable for distinguishing myxoid chondrosarcomas from chordoma, parachordoma, chordoid meningioma, chordoid glioma, metastatic carcinoma, and other myxoid neoplasms [9]. In addition, molecular genetic testing for EWSR1-associated translocations can provide further diagnostic support, particularly in anatomically unusual cases or when histopathologic findings are equivocal [7,8]. In the present case, however, immunohistochemical and molecular analyses were not available because of local resource limitations. Therefore, the diagnosis was based on routine histopathologic assessment in conjunction with the clinical and radiologic findings, and the absence of ancillary testing should be considered when interpreting the diagnosis. 

The approach to therapy for patients diagnosed with PICMCs involves maximal safe surgical resection as the fundamental aspect of treatment; however, achieving gross total resection is often severely compromised by constraints imposed by skull base anatomy, vascular encroachment, dural invasion, involvement of one or more of the lateral ventricles, and adhesion to functionally significant neural pathways [7-9]. Radiation therapy has frequently been employed postoperatively, especially in those patients who undergo less than a gross total resection or in whom the adequacy of resection margin(s) is questionable or who have bone involvement or anatomical constraints on resection [7,8]. The use of chemotherapy for PICMCs has been sparsely documented in the medical literature; temozolomide has been reported in some instances as having efficacy against certain intracranial cases; however, there is currently no standard chemotherapy protocol established for this tumor [8]. Due to the propensity for local recurrences and delayed progression with occasional distant metastasis, prolonged follow-up using clinical evaluation and imaging techniques is recommended regardless of whether the patient has undergone an apparent gross total resection.

The decision to pursue a transcranial approach rather than a conventional sellar approach was anatomically driven. The tumor's cephalad growth into the ventricular system and lateral extension rendered an endoscopic endonasal approach unsafe. This case highlights the principle that a surgical approach should be dictated by tumor extension, not location alone. The intraoperative findings of a cartilaginous, highly vascular tumor encasing the carotid artery and adherent to brain parenchyma explain the inability to achieve gross-total resection. Maximal safe resection was the appropriate surgical goal. The absence of an ultrasonic aspirator further limited internal debulking. Adjuvant radiotherapy (46 Gy) was appropriately delivered to the tumor bed following evidence of residual disease. The reduction in residual tumor size from 45x40 mm to 10x10 mm suggests a positive response to radiotherapy, although residual disease persists. Although the patient remained clinically stable, this is a limitation, particularly due to the loss of a patient to formal imaging surveillance and because myxoid chondrosarcomas have the risk of a late local recurrence.

This case should be interpreted in the context of several limitations. The pathological evaluation is limited due to the lack of diagnostic tools and resources available at the hospital where the patient received treatment. While the final diagnosis was provided, many other evaluations such as histological grade, immunohistochemistry, molecular analysis, and marginal status could not be included in this report, and therefore, we were unable to identify any additional characteristics regarding the biological nature of this tumor. Similarly, surgical treatment options were also constrained by resource availability at the hospital. Specifically, the absence of an ultrasonic aspirator made it difficult to perform controlled internal debulking. Consequently, the residual disease present after surgery should be viewed within the context of the maximum safely achievable resection strategy. Lastly, the post-surgery follow-up of this patient was limited by both patient-related logistical issues like geographical location and employment-related constraints. Although remote clinical follow-up was able to occur and the patient reported a resolution of symptoms, the recommended last post-operative MRI has not been performed.

## Conclusions

Primary intracranial tumors with histopathologic findings favoring myxoid chondrosarcomas are rare and should be considered in the differential diagnosis of atypical sellar and parasellar lesions, particularly when imaging demonstrates extensive extrasellar extension, ventricular compression, or other atypical features. Multimodal management, consisting of maximal safe surgical resection followed by adjuvant radiotherapy when appropriate, remains the cornerstone of treatment. In the present case, stable clinical and radiologic outcomes were achieved despite residual disease. However, immunohistochemical and molecular characterization, as well as histologic grading, Ki-67 proliferation index, and mitotic index assessment, were not available because of resource limitations, and this should be considered when interpreting the diagnosis. Long-term clinical and radiologic follow-up remains essential to monitor for recurrence or progression.

## References

[1] Brackmann DE, Teufert KB (2006). Chondrosarcoma of the skull base: long-term follow-up. Otol Neurotol.

[2] Bohman LE, Koch M, Bailey RL, Alonso-Basanta M, Lee JY (2014). Skull base chordoma and chondrosarcoma: influence of clinical and demographic factors on prognosis: a SEER analysis. World Neurosurg.

[3] Freda PU, Beckers AM, Katznelson L, Molitch ME, Montori VM, Post KD, Vance ML (2011). Pituitary incidentaloma: an endocrine society clinical practice guideline. J Clin Endocrinol Metab.

[4] Pamir MN, Ozduman K (2006). Analysis of radiological features relative to histopathology in 42 skull-base chordomas and chondrosarcomas. Eur J Radiol.

[5] Palmisciano P, Haider AS, Sabahi M (2021). Primary skull base chondrosarcomas: a systematic review. Cancers (Basel).

[6] Asa SL, Ezzat S (2009). The pathogenesis of pituitary tumors. Annu Rev Pathol.

[7] Zhu ZY, Wang YB, Li HY, Wu XM (2022). Primary intracranial extraskeletal myxoid chondrosarcoma: a case report and review of literature. World J Clin Cases.

[8] Qin Y, Zhang HB, Ke CS (2017). Primary extraskeletal myxoid chondrosarcoma in cerebellum: a case report with literature review. Medicine (Baltimore).

[9] Im SH, Kim DG, Park IA, Chi JG (2003). Primary intracranial myxoid chondrosarcoma: report of a case and review of the literature. J Korean Med Sci.

